# Comparative transcriptome profiling of amyloid precursor protein family members in the adult cortex

**DOI:** 10.1186/1471-2164-12-160

**Published:** 2011-03-24

**Authors:** Dorothee Aydin, Mikhail A Filippov, Jakob-Andreas Tschäpe, Norbert Gretz, Marco Prinz, Roland Eils, Benedikt Brors, Ulrike C Müller

**Affiliations:** 1 Department of Bioinformatics and Functional Genomics, Institute of Pharmacy and Molecular Biotechnology, Heidelberg University, Im Neuenheimer Feld 364, D-69120 Heidelberg, Germany; 2 Medical Research Center, Medical Faculty Mannheim, Heidelberg University, D-68167 Mannheim, Germany; 3 Institute for Neuropathology, University Hospital Freiburg, Freiburg, Germany; 4 Department of Theoretical Bioinformatics, German Cancer Research Center (DKFZ), D-69120 Heidelberg, Germany

## Abstract

**Background:**

The β-amyloid precursor protein (APP) and the related β-amyloid precursor-like proteins (APLPs) undergo complex proteolytic processing giving rise to several fragments. Whereas it is well established that Aβ accumulation is a central trigger for Alzheimer's disease, the physiological role of APP family members and their diverse proteolytic products is still largely unknown. The secreted APPsα ectodomain has been shown to be involved in neuroprotection and synaptic plasticity. The γ-secretase-generated APP intracellular domain (AICD) functions as a transcriptional regulator in heterologous reporter assays although its role for endogenous gene regulation has remained controversial.

**Results:**

To gain further insight into the molecular changes associated with knockout phenotypes and to elucidate the physiological functions of APP family members including their proposed role as transcriptional regulators, we performed DNA microarray transcriptome profiling of prefrontal cortex of adult wild-type (WT), APP knockout (APP^-/-^), APLP2 knockout (APLP2^-/-^) and APPsα knockin mice (APP^α/α^) expressing solely the secreted APPsα ectodomain. Biological pathways affected by the lack of APP family members included neurogenesis, transcription, and kinase activity. Comparative analysis of transcriptome changes between mutant and wild-type mice, followed by qPCR validation, identified co-regulated gene sets. Interestingly, these included heat shock proteins and plasticity-related genes that were both down-regulated in knockout cortices. In contrast, we failed to detect significant differences in expression of previously proposed AICD target genes including *Bace1*, *Kai1*, *Gsk3b*, *p53*, *Tip60*, and *Vglut2*. Only *Egfr *was slightly up-regulated in APLP2^-/- ^mice. Comparison of APP^-/- ^and APP^α/α ^with wild-type mice revealed a high proportion of co-regulated genes indicating an important role of the C-terminus for cellular signaling. Finally, comparison of APLP2^-/- ^on different genetic backgrounds revealed that background-related transcriptome changes may dominate over changes due to the knockout of a single gene.

**Conclusion:**

Shared transcriptome profiles corroborated closely related physiological functions of APP family members in the adult central nervous system. As expression of proposed AICD target genes was not altered in adult cortex, this may indicate that these genes are not affected by lack of APP under resting conditions or only in a small subset of cells.

## Background

Despite its key role in Alzheimer's disease (AD) pathogenesis, the physiological functions of the β-amyloid precursor protein (APP) and its close homologue, the β-amyloid precursor-like protein 2 (APLP2), are still poorly understood. This is due to two major problems complicating the in vivo analysis. i) APP is subject to complex proteolytical processing and ii) APP is part of a gene family with partially overlapping functions.

**Figure 1:**
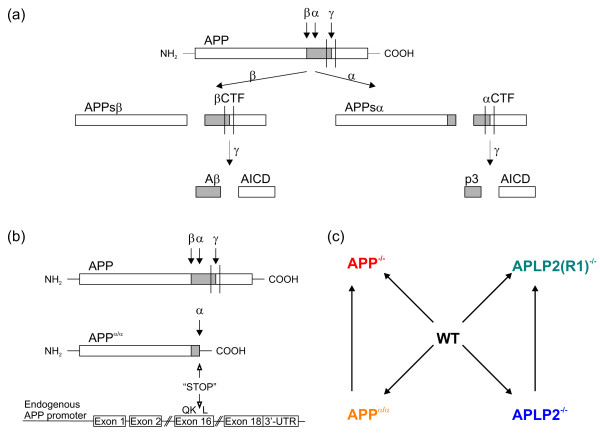
**Overview over study design**. (a) Overview of APP processing. APP is first cleaved by either α-secretase or β-secretase, thereby shedding soluble APPsα or APPsβ, respectively. The membrane-bound C-terminal fragments (CTFs) are then cleaved by γ-secretase: αCTF gives rise to p3 and AICD whereas βCTF is cleaved to Aβ and AICD. (b) In APP^α/α ^knock-in mice, a stop codon was introduced behind the α-secretase cleavage site by homologous recombination into the endogenous APP locus. Note that no full length APP or any other fragment can be generated from the APPsα knockin locus. (c) Overview of genotypes used for microarray analysis. APLP2(R1)^-/- ^had been backcrossed for one generation to C57BL/6 whereas WT, APP^-/-^, APP^α/α^, APLP2^-/- ^had been backcrossed for six generations. Six pairwise comparisons were performed: WT versus APP^-/-^, WT versus APP^α/α^, WT versus APLP2^-/-^, APP^α/α ^versus APP^-/-^, WT versus APLP2(R1)^-/-^, and APLP2^-/- ^versus APLP2(R1)^-/-^. The arrow indicates reference and tested group of each comparison.

APP is a type I transmembrane protein, and processing (see Figure 1a) is initiated either by α-secretase cleavage within the Aβ region, or by β-secretase (BACE) cleavage at the N-terminus of Aβ, leading to the secretion of large soluble ectodomains, termed APPsα and APPsβ respectively. Subsequent γ-secretase processing of the C-terminal fragments (βCTF, or αCTF) results in the production of secreted Aβ, p3 and the APP intracellular domain (AICD). Both APLPs are similarly processed by the same secretases. It is evident that APP/APLPs are highly complex molecules, that may exert important functions as unprocessed cell surface molecules (APP-FL) as well as functions mediated by their diverse proteolytic fragments. APP processing is highly reminiscent to that of Notch with γ-secretase-mediated release of the Notch intracellular domain (NICD) triggering the translocation of NICD to the nucleus. This results in transcriptional regulation of defined target genes involved in e.g. neuronal differentiation. Thus, a similar functional role for AICD (and the related intracellular fragments of APLPs, termed ALID1 and ALID2) as transcriptional regulator has been proposed [1]. Indeed, AICD has been shown to translocate to the nucleus and can form a complex with the adaptor FE65 and the histone acetyltransferase TIP60. This complex can induce the transcription of artificial reporter constructs in transfected cells [2,3]. Likewise, APLP1 and APLP2 are subject to γ-secretase processing and can stimulate the expression of heterologous reporter constructs in an FE65-dependent manner [4]. Additional complexity comes from recent studies indicating that APP (and both APLPs) can form tripartite complexes with the adaptor protein MINT3 and the transcriptional co-activators TAZ and YAP. When overexpressed in HEK293 cells, this complex functions in GAL4 reporter assays [5,6]. To date, several putative AICD target genes have been identified (mainly using overexpression approaches) including *Kai1 *[7], *Gsk3b *[8,9], *Nep *[10], *Egfr *[11], *p53 *[12], *Lrp *[13], *Tip60*, *Bace1*, *App *itself [14] as well as genes involved in cytoskeletal dynamics [15]. However, the validity of these proposed targets, in particular regarding the question of whether they also constitute endogenous AICD/ALID target genes, has remained controversial [16-22]. Interestingly, in several recent studies, increased production of AICD either in transfected cells or in transgenic animals did not lead to a consistent up-regulation of previously proposed target genes [15,20,22].

Previously, we showed that knockout (KO) mice deficient in a single family member such as APP (or one of the APLPs) are viable [23,24] whereas combined APP^-/-^APLP2^-/- ^or APLP1^-/-^APLP2^-/- ^double KO mice [24] and APP^-/-^APLP1^-/-^APLP2^-/- ^triple mutants [25] die shortly after birth, likely due to defects of neuromuscular transmission [26]. Neither APP^-/- ^nor APLP2^-/- ^mice display obvious defects of central nervous system (CNS) morphology, yet APP^-/- ^mice revealed reduced body weight and defects in spatial learning associated with impaired synaptic plasticity including long-term potentiation (LTP) [26]. However, the molecular mechanisms underlying these defects have remained unclear.

Processing of APP gives rise to several fragments including besides neurotoxic Aβ the α-secretase-generated soluble APPsα fragment that is neuroprotective and involved in synaptic plasticity [27,28]. To delineate its specific functions, we previously generated APPsα knockin (APP^α/α^) mice by inserting via gene targeting a stop codon into the endogenous APP locus right after the α-secretase cleavage site [28]. Thus, APP^α/α ^knockin mice express only secreted APPsα from the endogenous APP promoter (Figure 1b).

Here, we employed a rational unbiased approach and investigated transcriptional changes arising due to the lack of APP family members in the adult cortex of knockout mice to gain further insight into the physiological and signaling functions of APP family members. This includes transcriptome changes that may arise due to a lack of direct AICD/ALID-mediated transcriptional regulation as well as changes resulting from indirect signaling events mediated by transmembrane APP/APLP isoforms. First, we analyzed transcriptome changes due to the complete absence of APP or APLP2 (including all their proteolytic fragments) by conducting the pairwise comparisons of WT versus APP^-/- ^(WT/APP^-/-^) and WT versus APLP2^-/- ^(WT/APLP2^-/-^). Second, we had a closer look at the role of different APP fragments, in particular APPsα. Therefore, we compared the transcriptome of APP^α/α ^mice both to WT (WT/APP^α/α^) and APP^-/- ^mice (APP^α/α^/APP^-/-^), respectively. Third, we addressed the influence of the genetic background by comparing knockout animals of mixed 129 × C57BL/6 genetic background (APLP2(R1)^-/-^) to those backcrossed to C57BL/6 for 6 generations.

## Results and Discussion

We subjected prefrontal cortices of adult male mice (24 - 28 weeks of age) of the following groups to transcriptome analysis: WT (n = 3), APP^-/- ^(n = 3), APP^α/α ^(n = 3), APLP2^-/- ^(n = 3), APLP2(R1)^-/- ^(n = 3) (Figure 1c). WT, APP^-/-^, APP^α/α^, APLP2^-/- ^had been backcrossed for six generations to C57BL/6 mice. APLP2(R1)^-/- ^mice harbor the identical knockout allele as APLP2^-/- ^but were only backcrossed once. Note that APP^-/- ^mice lack membrane-anchored full length APP (APP-FL) as well as all proteolytic fragments derived from it (APPsα, Aβ, APPsβ, αCTF, βCTF and AICD), whereas APP^α/α ^mice express APPsα but lack full length APP and all other fragments.

Raw data was processed according to the RMA procedure [29,30]. We validated the microarray data by clustering the processed raw data based on all available *App*/*Aplp2 *probe sets. As expected, all samples grouped according to their genotypes: WT, APP^-/-^, APP^α/α^, and APLP2^-/- ^samples were clearly separated (Additional file 1).

### Differential gene expression in mice lacking APP family members

First, we wanted to address the question of which impact each genotype has on transcription by searching for genes that show differential expression in the different comparisons. To identify significantly up- or down-regulated genes, we performed a Significance Analysis of Microarrays (SAM) with a False Discovery Rate (FDR) of app. 5% (see Table 1 and Additional file 2).

**Table 1:** Overview over differentially expressed genes (R6 animals)

	total		up		down	
			
	FC≥2	no FCC	FC≥2	no FCC	FC≥2	no FCC
WT/APP^-/-^	**11**	359	**2**	274	**9**	85

WT/APLP2^-/-^	**11**	1242	**6**	1142	**5**	100

WT/APP^α/α^	**15**	447	**2**	250	**13**	197

APP^α/α^/APP^-/-^	**2**	29	**2**	29	**0**	0

The number of significant genes is displayed for all relevant comparisons if at least one probe set identifier of the gene meets the respective criteria. Note that probe sets for *App *and *Aplp2 *are not part of the indicated numbers. Numbers indicate significant genes either with fold change criterion (FC≥2, highlighted in bold) or without fold change criterion (no FCC). Note that all animals had been backcrossed for 6 generations to C57BL/6 (R6).

A total of 359 genes (274 up- and 85 down-regulated) were differentially expressed in WT/APP^-/-^. The comparison WT/APLP2^-/- ^led to 1242 differentially expressed genes (1142 up- and 100 down-regulated). For the comparison WT/APP^α/α^, we observed 447 significantly regulated genes (250 up- and 197 down-regulated). In contrast, we only observed 29 significant genes in the comparison APP^α/α^/APP^-/- ^(all of them were up-regulated). Based on the total number of significant differentially expressed genes in these comparisons, the APLP2 knockout has the highest impact on gene expression.

To get an idea how many differentially expressed genes with high fold changes are within each list, we introduced a fold change criterion and determined the number of genes that differ by at least 2-fold (Table 1). In the comparisons WT/APP^-/- ^11 genes, in WT/APLP2^-/- ^11 genes, in WT/APP^α/α ^15 genes, and in APP^α/α^/APP^-/- ^2 genes passed this criterion. This shows that the majority of significant differentially expressed genes show only small to moderate (up to 2-fold) alterations in gene expression. This is consistent with previous studies [31] and likely due to the complex nature of cortical tissue consisting of a multitude of neuronal and glial subpopulations. In APP^-/- ^and APP^α/α ^animals, no compensatory up-regulation of *Aplp1 *and *Aplp2 *at the mRNA level was observed. Likewise, no up-regulation of *App *and *Aplp1 *was observed in APLP2^-/- ^animals thus confirming previous Western blot results [24].

### Analysis of biological pathways affected in APP/APLP knockout mice

Subsequently, we analyzed the list of significant genes from all comparisons using DAVID bioinformatics resources [32]. Within DAVID, we did Functional Annotation Clustering using Gene Ontology terms (biological processes) and pathway databases (Biocarta, Reactome, Panther, KEGG) to gain an overview about the nature of genes and potential shared functional pathways.

**Figure 2:**
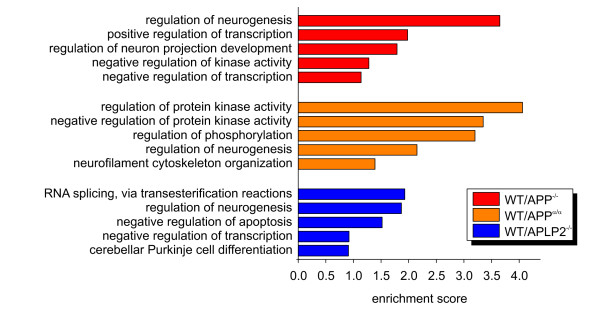
**Functional annotation clustering**. The five most enriched clusters in the comparisons WT/APP^-/-^, WT/APP^α/α^, WT/APLP2^-/- ^including their respective enrichment score are shown. The name of one gene group out of each cluster was taken to represent the complete cluster.

We found 24 enriched clusters in the comparison WT/APP^-/-^, 29 in WT/APP^α/α^, and 35 in WT/APLP2^-/- ^(Additional file 3). Due to the low number of significant genes in APP^α/α^/APP^-/-^, no gene set enrichment could be assessed. Interestingly, several of these enriched clusters were shared between the different pairwise comparisons including regulation of neurogenesis, transcription and kinase activity (Figure 2, Additional file 3).

The finding that lack of either APP or APLP2 affects expression of genes involved in neurogenesis confirms and further extends previous studies that implicated APP in neuronal progenitor regulation [33-35]. In APP-overexpressing transgenic mouse models, adult neurogenesis in the hippocampus is impaired [36] which has been mainly attributed to Aβ-mediated toxic effects. Regulation of transcription was identified as another shared cluster between WT/APP^-/- ^and WT/APLP2^-/- ^pointing towards similar functions of APP family members in this cellular process, possibly via AICD/ALID signaling or via more indirect mechanisms. Shared functional clusters were also found for WT/APP^-/- ^and WT/APP^α/α^, namely neurogenesis and negative regulation of protein kinase activity which may indicate that phenotypes of APP-KO mice, e.g. defects in synaptic plasticity, arise due to alterations in the phosphorylation state of yet to be identified target proteins.

Although Aβ serves as a central trigger for AD pathogenesis, the physiological role of APP and the question of whether a loss of its functions contributes to AD are still unclear. We therefore investigated a possible enrichment of genes previously linked to Alzheimer's disease in our dataset. Comparing the 359 genes differentially expressed in WT/APP^-/- ^with the AlzGene dataset (currently comprising 662 genes), we identified 14 genes, namely *Abcg4*, *Ache*, *Aldh2*, *Arsb*, *Bcl2*, *Bdnf*, *Crh*, *Egr2*, *Fos*, *Gstz1*, *Hspa1a*, *Hspa1b*, *Hspa5*, *Ppp1r3c*. Next, we assessed whether this number of 14 identified genes represents a significant enrichment of AD-related genes in the WT/APP^-/- ^dataset. To this end, we randomly drew 100 gene sets of the same size (n = 359) from the pool of genes covered by the array and checked them against the AlzGene set. We found an average of 9 genes per randomly drawn gene set and used this as reference for Fisher's exact test. However, no significant enrichment of genes from the AlzGene dataset was present in WT/APP^-/-^.

### Proposed AICD target genes show only a minor or no significant differential expression in APP- and APLP2-deficient cortex

**Figure 3:**
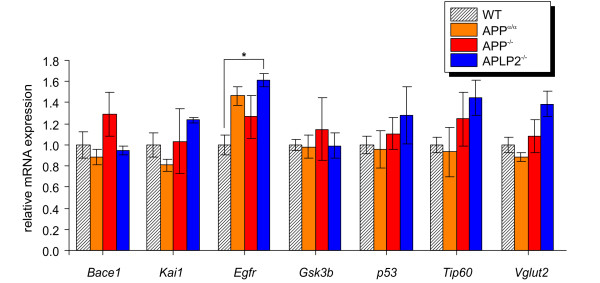
**qPCR analysis of indicated target genes**. mRNA expression was measured and displayed relative to wild-type level set as one. Values represent means ± SEM of 3 mice/genotype. (Student's t-test: *, p-value < 0.05).

Several genes have been proposed to be directly regulated at the transcriptional/promoter level by an AICD/FE65 transcriptionally active complex including *Bace1 *[14], *Kai1 *[7], *Egfr *[11], *Gsk3b *[8,9], *p53 *[12], *Tip60 *[14], and *Vglut2 *[37]. By array analysis we detected in APLP2^-/- ^mice for *Vglut2 *and *Gsk3b *a small yet significant up-regulation of 1.27-fold and 1.2-fold, respectively, compared to wild-type animals (Additional File 2). In all other genotypes, including APP^-/-^, expression was, however, not significantly altered. To further validate these results we conducted a qPCR analysis of these proposed target genes (Figure 3). This way, we detected a 1.6-fold increase of *Egfr *mRNA expression solely in APLP2^-/- ^animals, compared to wild type controls (p < 0.05). Of note, qPCR analysis of APP^-/-^, APP^α/α ^and APLP2^-/- ^cortex failed to detect significant expression differences of all other tested candidate genes, including *Vglut2 *and *Gsk3b*.

What might be the reasons that proposed target genes have proven difficult to confirm in follow-up studies including work reported here? A major reason may be the difference in experimental systems used as overexpression in cell lines may not necessarily reflect a role of AICD for endogenous gene expression. In line with this study, we previously found no impact on *Kai1*, *Gsk3a*, *Gsk3b*, *App*, and *Nep *mRNA expression when treating different cell lines with the γ-secretase inhibitor DAPT or when assessing endogenous gene expression in AICD-deficient model systems [16]. In addition, as shown by the same study, clonal variability of immortalized fibroblast lines may lead to variable gene expression irrespective of either APP^-/- ^or APP^+/+ ^genotype [16]. On the other hand, one might expect an inverse regulation of target genes upon AICD deficiency as opposed to overexpression. A genome-wide microarray-based approach to detect AICD target genes used an inducible FE65/AICD cell line [15]. Here, no change in *Kai1 *and *Gsk3b *mRNA expression was detected. Similarly, Waldron et al. [22] found no alteration in mRNA expression of *Kai1*, *Bace1*, *Egfr*, *Tip60*, and *p53 *in AICD-enriched FE65-tranfected cells. Moreover, transcriptome analysis in AICD transgenic mouse brain revealed no apparent difference between transgenic animals and littermate controls [20] and qPCR analysis of proposed target genes, including those studied here, failed to detect significant changes in mRNA expression. Overall, our results are highly consistent with these studies. Although our study clearly indicates that AICD or ALID2 are on their own not essential transcriptional regulators of tested target genes in adult prefrontal cortex, we cannot exclude at present that other APP family members (including APLP1) may at least partially compensate for a single gene deficiency. Due to the lethality of combined mutants shortly after birth we had previously analyzed the expression of a subset of target genes in APP^-/-^APLP2^-/- ^embryonic brain and fibroblasts [16]. As APLP1 is not expressed in fibroblasts, APP^-/-^APLP2^-/- ^fibroblasts (compared to APP retransfected cells) provide a cellular model in which all APP family members are lacking. However, neither *Nep *nor *Gsk3b *expression was significantly affected in either embryonic brain or fibroblasts [16]. A global assessment of transcriptome changes in adult brain lacking multiple APP family members (a tissue more relevant for AD) will await the generation of viable conditional mutants. Considering the complexity of cortical tissue, it is still possible that gene expression differences occurring only in distinct cell types may remain below the detection limit of our analysis. In line with this hypothesis, Schrenk-Siemens et al [37] reported a reduction of *Vglut2 *mRNA and VGLUT2 protein expression in glutamatergic neurons obtained by retinoic acid differentiation of APP^-/-^APLP2^-/- ^embryonic stem cells whereas in this study no difference was detectable in cortical tissue. It remains to be seen whether regulation of other target genes might also be cell type-specific.

### Genes co-regulated due to the lack of either APP or APLP2

**Figure 4:**
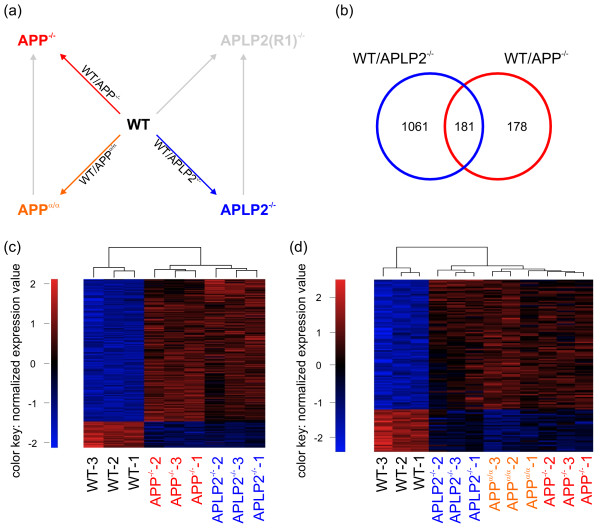
**Co-regulation of genes in WT/APP^-/- ^and WT/APLP2^-/-^**. (a) Overview of the analyzed comparisons. (b) Venn diagram of the two comparisons WT/APP^-/- ^and WT/APLP2^-/- ^based on gene lists obtained by significance analysis. The number of significant genes is indicated in the respective segments. Note that 181 genes are co-regulated by the lack of either APP or APLP2. (c) Heatmap of the 213 identifiers corresponding to the set of 181 genes that are co-regulated in both pairwise comparisons. All identifiers show differential expression in the same direction. (d) Heatmap of 122 identifiers corresponding to the set of 97 genes that are found in both pairwise comparisons as well as in WT/APP^α/α^. The values of the heatmaps (c,d) are normalized expression values with red and blue color representing the number of standard standard deviations above or below the mean expression for each probe set, respectively.

As there is genetic [24,25] and cell biological evidence (such as a shared set of protein interaction partners [38]) that APP family members serve related physiological functions, we searched for genes that are, compared to wild-type, differentially regulated in more than one genotype (Figure 4a). To this end, we created a Venn diagram of the pairwise comparisons WT/APP^-/- ^and WT/APLP2^-/-^. We found 213 probe set identifiers (Additional file 4) representing 181 known genes that are differentially expressed in both cases (Figure 4b) and regulated in the same direction as shown by cluster analysis (Figure 4c). Furthermore, we analyzed how many of these genes were in addition significantly regulated in WT/APP^α/α^. Out of 181 genes co-regulated by lack of either APP or APLP2, 97 were also found in the comparison WT/APP^α/α ^and regulated in the same direction (Figure 4d, Additional file 4). Thus, functional similarities of APP family members are also reflected at the transcriptional level by co-regulated gene sets in the respective loss-of-function mutants. To investigate this more closely we further examined two of these genes by qPCR analysis: heat shock protein 5 (*Hspa5*) and cyclin-dependent kinase inhibitor 1A (*Cdkn1a*).

**Figure 5:**
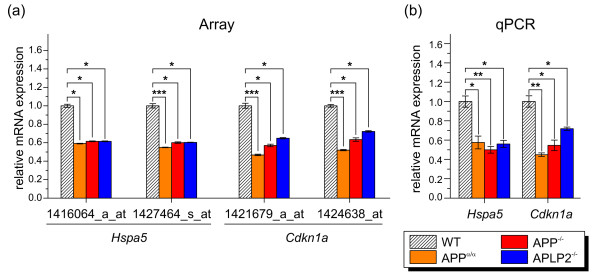
**Relative mRNA expression of selected genes co-regulated in all comparisons**. (a) Relative mRNA expression obtained by array analysis. Values of indicated probe set identifiers were compared to WT levels. Values represent relative expression value ± relative standard deviation. Differences were tested for significance by SAM (*, q-value < 0.05; **, q-value < 0.01; ***, q-value < 0.001). (b) The corresponding mRNA expression was measured by qPCR and displayed relative to wild-type level set as one. Values represent means ± SEM of 3 animals per group. (Student's t-test: *, p-value < 0.05; **, p-value < 0.01; ***, p-value < 0.001).

*Hspa5 *attracted our attention for two reasons. Amongst co-regulated genes we found a consistent down-regulation of four heat-shock proteins including besides *Hspa5*, *Hspa1b*, *Hspb1*, and *Hsph1*. HSPA5 (also known as GRP78) is an ER chaperone involved in the ER stress response and had previously been shown to interact with APP and modulate Aβ production [39]. In addition, GRP78 was recently identified as a gene that may counteract the proliferative effect of secreted APPs in tumor models [40]. As functional annotation clustering identified neurogenesis as a pathway affected in all genotypes compared to wild-type, it was interesting to find *Cdkn1a*, also known as *p21*, amongst these co-regulated genes. Based on array analysis, both *Cdkn1a *and *Hspa5 *were down-regulated by about two-fold in all three mutant genotypes compared to WT (Figure 5a). Employing qPCR we confirmed these results and found again a significant down-regulation of about the same magnitude (Figure 5b).

It is noteworthy that in APP-overexpressing transgenic mice *Hspa5 *had previously been found to be up-regulated [41] suggesting an inverse transcriptional regulation as a consequence of either loss or gain of APP-dependent signaling. The CDK inhibitor p21 has been shown to restrict adult neurogenesis in the hippocampus, as evidenced by increased proliferation of neuronal progenitors in p21^-/- ^mice [42]. Given the *Cdkn1a/p21 *down-regulation we found here, one might thus expect APP^-/- ^(or APLP2^-/-^) mice to show dysregulated neurogenesis. On the other hand, we had previously shown that endogenous APPs and APLP2s play a crucial role as growth factors for neuronal stem cells in the adult subventricular zone (SVZ) [35]. Depletion of APPsα by infusion of APP-binding antibodies or as a consequence of pharmacological inhibition of APPsα production reduced the number of neuronal progenitor cells in the SVZ [35]. Thus neurogenesis might be under complex control of APP-mediated signaling pathways, both by membrane-anchored APP and secreted APP isoforms.

### Role of APP domains for transcriptome changes

**Figure 6:**
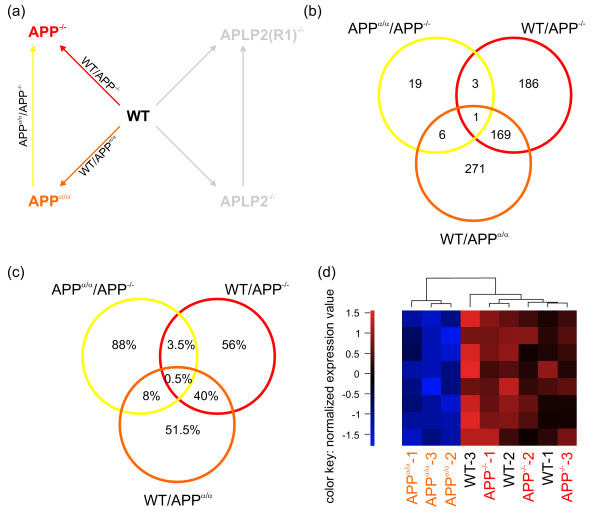
**Analysis of common genetic profiles in WT/APP^-/-^, WT/APP^α/α ^and APP^α/α^/APP^-/-^**. (a) Overview of the analyzed comparisons. (b) Venn diagram with SAM-based gene lists of the three comparisons WT/APP^-/-^, WT/APP^α/α ^and APP^α/α^/APP^-/-^. The numbers indicate the absolute number of differentially expressed genes in the respective comparison. (c) Percentage of probe set identifiers co-regulated in two or three pairwise comparisons. Values are based on the 200 most significantly differentially expressed identifiers for each pairwise comparison. Note that 40% of probe sets are found in the overlap between WT/APP^-/- ^and WT/APP^α/α^. (d) Heatmap of the probe set identifiers corresponding to the set of 6 genes (b) in the intersection of WT/APP^α/α^, APP^α/α^/APP^-/-^. The values of the heatmap are normalized expression values with red and blue color representing the number of standard standard deviations above or below the mean expression for each probe set, respectively.

Next, we compared the transcriptome of APP^-/- ^mice to that of APP^α/α ^animals (Figure 6a). Specifically, we wanted to answer the question of whether the transcriptome of APP^α/α ^mice would be more similar to that of WT mice (which would indicate that APP-FL and/or the APP C-terminus is of minor importance), or would rather resemble that of APP^-/- ^cortices (which would indicate an important role of APP-FL and/or APP C-terminal fragments for signaling). As APPsα is sufficient to rescue the learning impairment and LTP defect of APP^-/- ^mice [28], we initially inferred that this would also apply for the transcriptome of APP^α/α ^mice. However, in the significance analysis, APP^α/α^/APP^-/- ^was the comparison with the lowest number of significant genes whereas the number of significant differentially expressed genes for WT/APP^-/- ^and WT/APP^α/α ^was at least 10 times higher (see Table 1) indicating a close resemblance of APP^α/α ^and APP^-/- ^samples. Next, we generated a Venn diagram to gain an overview on the absolute number of significant genes found in each intersection (Figure 6b, Additional file 5). A high proportion of genes significant in WT/APP^α/α ^was also significant in WT/APP^-/- ^leading to a subset of 169 co-regulated genes. Second, to get a more quantitative understanding on this part of the dataset, we calculated the percentage of probe sets that were co-regulated between the different pairwise comparisons. To this end, we ranked all probe sets according to their absolute significance score and used the most significant 200 probe sets of each of these pairwise comparisons to generate the Venn diagram (Figure 6c). WT/APP^-/- ^and WT/APP^α/α ^have 40% of the 200 probe sets in common whereas WT/APP^-/- ^and APP^α/α^/APP^-/- ^share only 7 out of 200 probe sets (Figure 6c). Taken together these results suggest that with regard to transcriptional changes APP^α/α ^is functionally very similar to a complete APP knockout. These data may indicate an important role of full length APP and/or APP C-terminal fragments for direct or indirect signaling resulting in transcriptome changes.

In addition, we raised the question whether the constitutive expression of APPsα would lead to changes in gene expression, as recently reported for APPsβ [43]. We found a small percentage (8.5%) of probe sets in the intersection of APP^α/α^/APP^-/- ^and WT/APP^α/α ^(Figure 6c). To investigate this finding further, we analyzed the 6 significant genes that are found within this group (Figure 6b) and used their corresponding 8 probe sets for a cluster analysis (Figure 6d). Hierarchical clustering results in a clear separation of APP^α/α ^from WT and APP^-/- ^cortices. This points to a small subpopulation of genes that are actually regulated by the constant production of APPsα in the absence of APP full length and all other fragments.

**Figure 7:**
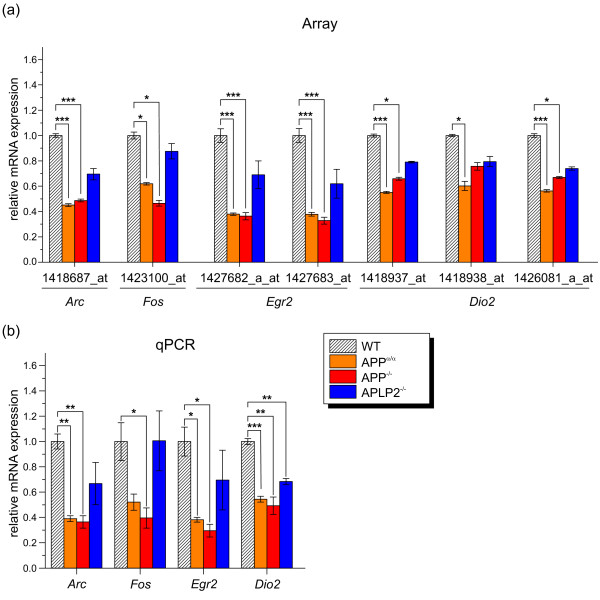
**Relative mRNA expression of selected genes co-regulated in WT/APP^-/- ^and WT/APP^α/α^**. (a) Relative mRNA expression level obtained by array analysis. Values of indicated probe set identifiers/genes were compared to WT levels. Values represent relative expression value ± relative standard deviation. Differences were tested for significance by SAM (*, q-value < 0.05; **, q-value < 0.01; ***, q-value < 0.001). (b) The corresponding mRNA expression was measured by qPCR and displayed relative to wild-type level set as one. Values represent means ± SEM of 3 animals per group. (Student's t-test: *, p-value < 0.05; **, p-value < 0.01; ***, p-value < 0.001).

Next, we took a closer look at genes co-regulated by the complete absence of APP or APP-FL as identified in the intersection of WT/APP^-/- ^and WT/APP^α/α ^(Figure 6b). Interestingly, this gene set comprises several synaptic plasticity-related genes including the immediate early response factors *Arc *(activity-regulated cytoskeleton-associated protein), *Fos *(FBJ osteosarcoma oncogene), *Egr2 *(early growth response 2), and *Dio2 *(deiodinase, iodothyronine, type II), a key enzyme in the biosynthesis of the nuclear hormone Triiodothyronine (T3). Validation of these genes by qPCR (Figure 7) consistently identified an about 1.5- to 2-fold down-regulation in all genotypes and thus confirmed gene expression changes identified by array analysis. In case of *Dio2*, down-regulation in APLP2^-/- ^now reached significance level when using qPCR analysis as compared to array values.

*Arc *mRNA accumulates in activated synapses, modulates AMPAR trafficking and is critically involved in memory consolidation and LTP [44]. Both FOS, best known for its binding to the Jun/AP-1 transcription factor complex, and the Zn^2+^-finger transcription factor EGR2/KROX-20 are induced during neuronal activity [45,46] and play an important role in learning and memory as well as LTP [46-48]. As APP^-/- ^mice show an age-dependent deficit in spatial learning associated with impaired long-term potentiation (LTP), it was intriguing that we found a down-regulation of genes previously implicated in synaptic plasticity although further studies are needed to establish a causal link.

### Influence of genetic background on gene transcription

**Figure 8:**
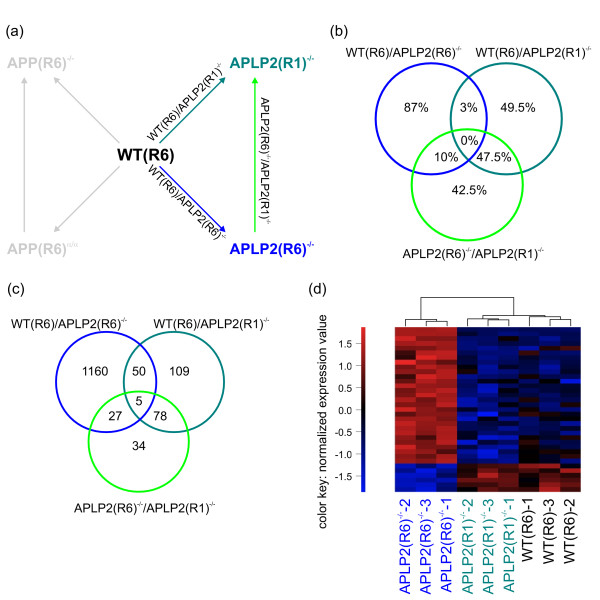
**Co-regulation of genes in WT(R6)/APLP2(R6)^-/-^, WT(R6)/APLP2(R1)^-/-^, APLP2(R6)^-/-^/APLP2(R1)^-/-^**. (a) Overview of the analyzed comparisons. (b) Percentage of probe set identifiers co-regulated in two or three pairwise comparisons. Values are based on the 200 most significantly differentially expressed identifiers for each pairwise comparison. (c) Venn diagram with SAM-based gene lists of the three comparisons WT(R6)/APLP2(R6)^-/-^, WT(R6)/APLP2(R1)^-/- ^and APLP2(R6)^-/-^/APLP2(R1)^-/-^. (d) Cluster analysis of probe sets corresponding to the set of 27 genes from (c) found exclusively in the comparisons WT(R6)/APLP2(R6)^-/- ^and APLP2(R6)^-/-^/APLP2(R1)^-/-^. The values of the heatmap are normalized expression values with red and blue color representing the number of standard standard deviations above or below the mean expression for each probe set, respectively.

Genetic background is known to profoundly influence the occurrence, penetrance and severity of transgenic and knockout phenotypes, e.g. with regard to behavior or Aβ deposition [49,50]. For one of our mutants, APLP2^-/-^, we had kept animals that had been backcrossed only once to C57BL/6 (designated APLP2(R1)^-/-^), whereas all other mutants had been backcrossed for six generations (designated R6). This allowed us to investigate the impact of genetic background on transcriptome changes versus changes that arise as a consequence of APLP2 gene deficiency (Figure 8a). To make this more clear for the reader, we will for the remaining study use the designation R6 and R1 for all subsequent comparisons (please note however that in figures 1,2,3,4,5,6,7 all animals were also of R6 genetic background).

**Figure 9:**
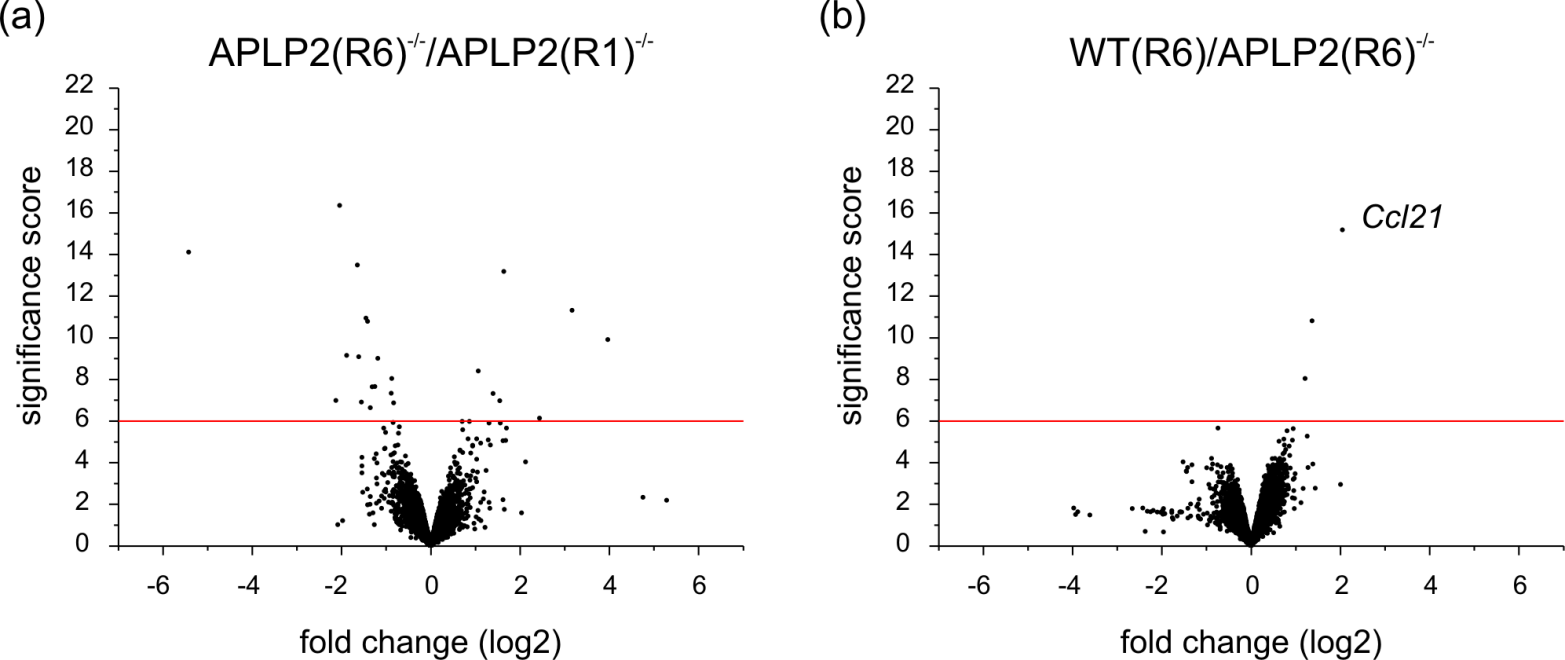
**Volcano plot of APLP2(R6)^-/-^/APLP2(R1)^-/- ^and WT(R6)/APLP2(R6)^-/-^**. The absolute value of each probe set identifier's significance score was plotted against the corresponding log2-transformed fold change of (a) APLP2(R6)^-/-^/APLP2(R1)^-/- ^and (b) WT(R6)/APLP2(R6)^-/-^. The red line was set arbitrarily to a score value of 6 to highlight the difference between the two Volcano plots. (APLP2-specific data points were removed prior to plotting).

The comparison APLP2(R6)^-/-^/APLP2(R1)^-/-^, i.e. between animals of the same genotype but with different genetic background, led to 144 significant differentially expressed genes of which 50 were up- and 94 were down-regulated (Table 2, Additional file 6). For the comparison WT(R6)/APLP2(R1)^-/- ^(animals with both different genotype and different genetic background) we found a total of 242 genes, 157 up-, 85 down-regulated (Table 2, Additional file 6). The sizes of these two sets are more than 5-fold smaller than the 1242 genes found for WT(R6)/APLP2(R6)^-/- ^(Table 2, Additional file 6). However, genetic background-related probe sets differentially regulated between APLP2(R6)^-/- ^versus APLP2(R1)^-/- ^have very high significance scores due to high gene expression changes (Additional file 6). This considerable influence of genetic background is best reflected in the corresponding volcano plot (Figure 9) yielding, when compared to WT(R6)/APLP2(R6)^-/- ^(Figure 9b), a much larger number of probe sets with both high significance score (arbitrarily set to 6) and high fold change in the comparison APLP2(R6)^-/-^/APLP2(R1)^-/- ^(Figure 9a). Of note, only three probe sets matching these criteria were found for the comparison WT(R6)/APLP2(R6)^-/- ^(Figure 9b). Similarly, this also holds true for any of the other pairwise comparisons for which genetic background was kept constant (data not shown).

**Table 2:** Overview over differentially expressed genes: impact of genetic background

	total		up		down	
			
	FC≥2	no FCC	FC≥2	no FCC	FC≥2	no FCC
WT(R6)/APLP2(R6)^-/-^	**11**	1242	**6**	1142	**5**	100

WT(R6)/APLP2(R1)^-/-^	**35**	242	**19**	157	**16**	85

APLP2(R6)^-/-^/APLP2(R1)^-/-^	**40**	144	**19**	50	**21**	94

The number of significant genes is displayed for all relevant comparisons if at least one probe set identifier of the gene meets the respective criteria. Note that probe sets for *App *and *Aplp2 *are not part of the indicated numbers. Numbers indicate significant genes either with fold change criterion (FC≥2, highlighted in bold) or without fold change criterion (no FCC). R6: backcrossed to C57BL/6 for 6 generations. R1: backcrossed to C57BL/6 for 1 generation. Note that the comparison WT(R6)/APLP2(R6)^-/- ^is identical to WT/APLP2^-/-^.

To study the impact of genetic background more closely, we calculated the percentages of overlap from the most significant 200 probe sets for each of the three comparisons (Figure 8b). If transcriptome changes arise primarily as a consequence of APLP2 deficiency independent of genetic background, we would expect a high number of co-regulated probe sets in the comparisons WT(R6)/APLP2(R6)^-/- ^and WT(R6)/APLP2(R1)^-/-^. The percentage of probe sets found in this intersection is, however, surprisingly small (3%). Contrary to our expectation, the highest overlap of probe sets (47.5%) is found in the intersection of WT(R6)/APLP2(R1)^-/- ^and APLP2(R6)^-/-^/APLP2(R1)^-/- ^(Figure 8b).

To gain an overview on absolute number of differentially expressed genes, we created a Venn diagram from the pairwise comparisons WT(R6)/APLP2(R6)^-/-^, WT(R6)/APLP2(R1)^-/-^, and APLP2(R6)^-/-^/APLP2(R1)^-/- ^(Figure 8c, Additional file 7). Interestingly, we found a set of 27 significant genes in the intersection of WT(R6)/APLP2(R6)^-/- ^and APLP2(R6)^-/-^/APLP2(R1)^-/- ^(Figure 8c). The corresponding cluster analysis shows that APLP2(R1)^-/- ^samples cluster together with WT(R6) samples while APLP2(R6)^-/- ^samples were clearly separated (Figure 8d). Probe sets in this intersection represent genes that are differentially expressed due to APLP2 deficiency but only in combination with an R6 background. In summary, these findings clearly indicate that genetic background may dominate transcriptome changes and needs to be carefully controlled to establish a clear link between phenotypes and altered genotype.

**Figure 10:**
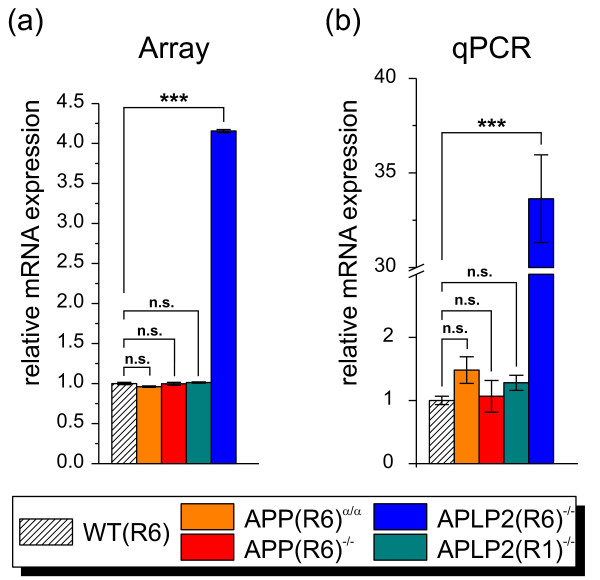
**Relative mRNA expression of *Ccl21***. (a) Array-based analysis of *Ccl21 *mRNA expression normalized to wild-type level. Values represent relative expression value ± relative standard deviation. Significance was tested by SAM (*, q-value < 0.05; **, q-value < 0.01; ***, q-value < 0.001). (b) qPCR analysis of *Ccl21 *mRNA expression displayed relative to wild-type level set as one. Values represent means ± SEM. (Student's t-test,*, p-value < 0.05; **, p-value < 0.01; ***, p-value < 0.001).

**Figure 11:**
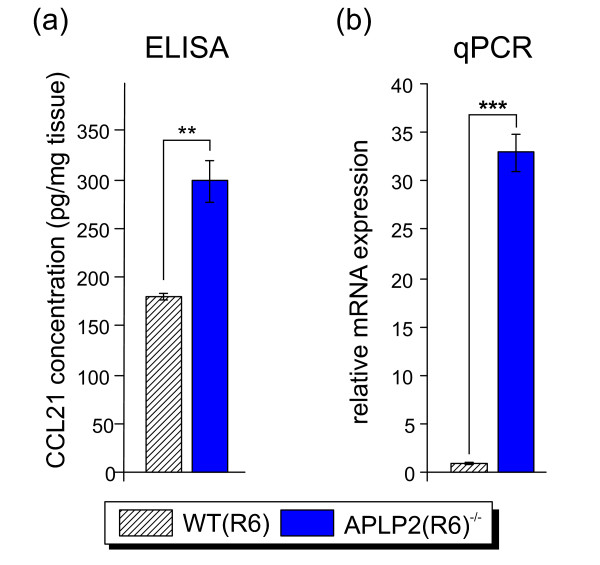
**Analysis of CCL21 protein and *Ccl21 *mRNA expression**. (a) CCL21 protein expression was determined by ELISA in either WT(R6) (n = 3) or APLP2(R6)^-/- ^(n = 4) brain tissue from one hemisphere comprising cortex, hippocampus and olfactory bulb. (b) qPCR analysis of *Ccl21 *mRNA expression using brain tissue from the contralateral side of the same animals (identical brain regions as in (a). (Student's t-test;*, p-value < 0.05; **, p-value < 0.01; ***, p-value < 0.001).

Eventually, we were interested to identify genes that are highly differentially expressed in our knockout models compared to wild-type. *Ccl21 *(chemokine (C-C motif) ligand 21) was the gene with the highest fold change in combination with the highest significance score and exclusively up-regulated in APLP2^-/- ^cortices on R6 genetic background (Figure 10a), that is preferred in behavioral studies and to which we had therefore backcrossed our mutants. In the periphery, CCL21 serves as ligand for CCR7 that is expressed by various cells of the immune systems and is involved in lymphocytes homing (reviewed in [51]). Recent studies show that CCL21 is also expressed in the CNS by endangered neurons to activate microglia via CXCR3 [52,53]. CCL21 is transported in vesicles along the axon to presynaptic structures and thereby constitutes a mediator of directed neuron-microglia signaling and remote microglia activation [54]. As microglia activation is associated with AD pathogenesis [55] and is also frequently observed as a general indicator of brain damage, we investigated CCL21 expression in more detail. Moreover, we sought to validate the genetic background dependence of CCL21 expression. Using qPCR analysis, we confirmed the significant up-regulation of *Ccl21 *mRNA yielding a fold change in qPCR (33-fold up-regulation) that was even more pronounced compared to array analysis (4-fold up-regulation) (Figure 10b). Importantly and consistent with our array analysis, no up-regulation of *Ccl21 *mRNA expression was found in APLP2(R1)^-/- ^samples. This indicates that i) other loci distinct from APLP2 are involved in *Ccl21 *transcriptional regulation and that ii) these loci give rise to allelic variants that differ functionally between the R6 and R1 genetic background. Encouraged by the high increase of *Ccl21 *mRNA expression, we determined CCL21 protein expression by ELISA in cortical tissue of APLP2(R6)^-/- ^animals and wild-type controls. CCL21 protein expression was significantly increased by about 1.7-fold (Figure 11a). As this magnitude of up-regulation was lower than expected from qPCR (Figure 10b), we also measured mRNA levels of *Ccl21 *in the same brain samples used for the ELISA measurement and reconfirmed the high up-regulation of about 33-fold (Figure 11b). This difference in differential mRNA (33-fold) and protein (1.7-fold) expression suggests that additional posttranslational mechanisms (including e.g. protein stability and turnover) limit CCL21 expression. In line with the moderate induction of CCL21 protein expression we did not detect an increase in gliosis in APLP2-deficient brains using immunohistochemistry (data not shown).

## Conclusions

Here, we determined the effect of APP^-/-^, APP^α/α ^and APLP2^-/- ^genotypes on gene expression in the adult murine cortex. We found large sets of differentially expressed genes, however, fold changes were in most cases only small to moderate. Previously proposed AICD target genes were not convincingly affected by lack of either APP or APLP2 (and thus lack of AICD and ALID) in the complex cortical tissue of adult brain. This may either indicate that the role of AICD in transcriptional regulation has been overestimated or that gene expression changes occur only in a distinct subset of cells that is below the detection level of our analysis.

Remarkably, we found the largest set of differentially expressed genes in APLP2^-/- ^brain, although so far no apparent morphological or other phenotypic changes had been reported for APLP2-KO mice. A substantial proportion of genes were identified as co-regulated by lack of APP or APLP2, notably in pathways such as neuronal differentiation, neurogenesis and transcriptional regulation. This common genetic profile points towards shared physiological functions in these pathways. When comparing APPsα knockin mice and APP^-/- ^mice we observed a close resemblance of the two genotypes pointing towards a crucial role of the APP C-terminus for transcriptome changes. Interestingly, we could demonstrate that several synaptic plasticity-related genes found in this gene set are considerably down-regulated which further substantiates the importance of APP family members in this regard.

Finally, we addressed the role of genetic background for transcriptome changes. Here, we report that the presence of different WT-alleles can lead to profound changes in gene expression that are even higher in magnitude than those resulting from the knockout of a single gene such as APLP2. Thus, it is crucial to keep genetic background constant, particularly if gene expression changes are rather subtle to reliably correlate affected pathways (and physiological functions inferred from them) with a knockout phenotype. In many studies regarding AICD signaling this issue has not been addressed which may at least partially explain the conflicting results reported by different laboratories. Here, we identified the chemokine *Ccl21 *as a gene that is highly up-regulated in APLP2^-/- ^cortex, but only in conjunction with C57BL/6-specific background alleles. Moreover, our study corroborates that APP family members are not only structurally related but also serve related physiological functions. It will therefore be of high interest to analyze phenotypic and gene expression changes in adult APP/APLP2 double or APP/APLP1/APLP2 triple deficient brain, once viable conditional combined mutants become available that are currently generated by crossing mice with floxed APP and APLP2 alleles with transgenic tissue-specific Cre mice [56].

## Methods

### Data

Raw and processed data discussed in this publication have been deposited in the NCBI's Gene Expression Omnibus database (GEO) and are accessible through GEO Series accession number GSE25926 (http://www.ncbi.nlm.nih.gov/geo/query/acc.cgi?acc=GSE25926).

### Animals

APP^-/-^, APLP2^-/-^, APP^α/α ^animals were previously described [28,57,58]. All animals were kept under specific pathogen free housing conditions (SPF unit) and in compliance with the regulations of the German animal protection law. For transcriptome analysis, animals had been backcrossed to C57BL/6 wild-type animals for 6 generations (R6) before they were interbred to homozygosity. All animals were adult males (24-28 weeks) and not challenged with any cognitive or stress tasks.

### RNA preparation and microarray data generation

Animals were sacrificed by cervical dislocation. Mouse brains were dissected and stored in RNAlater (Qiagen) at -20°C. Subsequently, the prefrontal cortex was cut out and used for total RNA preparation (RNAeasy kit, Qiagen). Quality of RNA was assessed with a spectrophotometer and Bioanalyzer (Agilent). 1 μg of total RNA was used for cDNA preparation (Oligo(dT) method, Invitrogen). Subsequent cRNA was prepared with Affymetrix One-Cycle Target Labeling and Control Reagent kit (Affymetrix Inc., Santa Clara, California, USA). The biotinylated cRNA was hybridized onto GeneChip Mouse Genome 430 2.0 Arrays (Affymetrix, Santa Clara). Chips were washed and scanned on the Affymetrix Complete GeneChip^® ^Instrument System generating digitized image data files.

### Statistical analysis

If not stated otherwise, data analysis and processing was carried out within the statistical computing environment R, version 2.8.0, using Bioconductor, BioC Release 2.4 [59]. Raw data was processed with the RMA algorithm (Robust Multiarray Average) developed by Irizarry et al. [29] and normalized using quantile normalization [30].

Hierarchical clustering was carried out using Euclidean distances to calculate the distances between the genes and between the sample groups. Calculated distances were clustered by complete linkage clustering. Expression values for each probe set were normalized to zero mean and unit variance. The values shown thus represent the number of standard deviations above or below the mean expression for each gene. Calculated expression differences for each probe set can be found in the respective additional file.

Significant differentially expressed probe sets between two groups were detected by a Significance Analysis of Microarrays (SAM) [60]. As a cut-off value for significance, we set the false discovery rate (FDR) to 5.33% (WT/APP^-/-^), 4.96% (WT/APLP2^-/-^), 4.5% (WT/APP^α/α^), 4.79% (APP^α/α^/APP^-/-^), 5.02% (WT(R6)/APLP2(R1)^-/-^), and 5.11% (APLP2(R6)^-/-^/APLP2(R1)^-/-^).

For counting significant differentially expressed genes, probe set identifiers were mapped to Entrez Gene identifiers. If at least one probe set was significant in the SAM, the gene was regarded to be significant as well. If no gene information (Entrez ID) was available for a certain probe set, the probe set was not counted.

For group testing (GO terms, pathways) DAVID bioinformatics resources was used [61]. Gene symbols from each list were taken as input, and redundant entries were discarded. The following gene sets were included into the analysis: GOTERM_BP_FAT (Gene Ontology), Biocarta (Pathways), KEGG_PATHWAY, PANTHER_PATHWAY, REACTOME_PATHWAY. Functional annotation clustering was carried out using the highest classification stringency.

### Quantitative real-time PCR (qPCR)

Total RNA was prepared using High Capacity cDNA kit based on random hexamer primer method (Applied Biosystems). For each qPCR reaction 20ng of total RNA were reverse transcribed into cDNA. qPCR was performed using FAM™-MGB dye labeled TaqMan^® ^Gene Expression Assays (Applied Biosystems) for *Bace1 *(assay Mm00478664_m1), *Kai1 *(assay Mm00492061_m1), *Egfr *(assay Mm01187858_m1), *Gsk3b *(assay Mm00444911_m1), *p53 *(assay Mm01731290_g1), *Tip60 *(assay Mm00724374_m1), *Vglut2 *(assay Mm00499876_m1), *Hspa5 *(assay Mm00517690_g1), *Cdkn1a/p21 *(assay Mm01303209_m1), *Arc *(assay Mm00479619_g1), *Fos *(assay Mm00487426_g1), *Egr2 *(assay Mm00456650_m1), *Dio2 *(assay Mm00515664_m1), *Ccl21 *(assay Mm03646971_gH) and beta-Actin as an internal standard (assay 4352933E). Quantification of qPCR results were evaluated by the 2^-ΔΔCT ^method and normalized to wild-type animals. Significance was calculated using unpaired Student's t-test (*, p < 0.05; **, p < 0.01; ***, p < 0.001).

### CCL21 ELISA measurements

Brain homogenates for ELISA were generated as described before [62]. Briefly brains were homogenized in lysis buffer (100mM phosphate, pH 7.4, 1mM EDTA, supplemented with complete protease inhibitor cocktail (Roche, Germany) using a PotterS homogenizer (Sartorius, Germany), followed by centrifugation at 1,500 ×g for 10 min. Supernatants were directly used for ELISA determinations.

ELISA measurements were performed using a mouse CCL21/6Ckine kit (R & D Systems Inc., MN) according to the manufacturer's protocol with slight modifications. Briefly, the standard curve was performed in a concentration range of 0 - 5000 pg/ml, the antibodies were used in the dilutions suggested by the protocol, except for the HRP-streptavidine conjugate, which was diluted 1:100. As a substrate one-step TMB-ELISA (Thermo Scientific, IL) was used.

## Authors' contributions

All authors read and approved the final manuscript. DA performed the bioinformatics analysis and drafted the manuscript. MAF harvested the tissue, performed all molecular genetics experiments and co-drafted the manuscript. JAT performed ELISA experiments. NG coordinated and supervised microarray hybridization and data acquisition. MP performed immunohistochemistry of brain sections to assess gliosis. RE coordinated the bioinformatics analysis. BB participated in study design, supervised the bioinformatics analysis and contributed to drafting the manuscript. UCM conceived and designed the study and drafted the final version of the manuscript.

## Supplementary Material

Additional file 1 **Heatmap of the processed dataset**. The heatmap shows the clustering of the processed data by *App*/*Aplp2*-specific probe sets. *App *and *Aplp2 *probe sets were taken from the ENSEMBL database and remapped onto the modified respective genomic loci of APP^-/-^, APP^α/α^, and APLP2^-/- ^animals. Only probe sets that map to exonic sequences or UTRs were chosen for hierarchical cluster analysis. The first three probe sets correspond to *App *probe sets whereas the last five are *Aplp2*-specific. The values of the heatmap are normalized expression values with red and blue color representing the number of standard standard deviations above or below the mean expression for each probe set, respectively. Click here for file

Additional file 2 **Lists of significant probe sets (R6 animals) resulting from the pairwise significance analyses**. for each significant Affymetrix probe set identifier, information about the gene (ENTREZ gene ID, gene symbol, gene name) and the output from the SAM (score, numerator, denominator, fold change, q-value) are displayed. Data was ranked by the absolute test score. WT/APP^-/- ^(= WTAPP), WT/APLP2^-/- ^(= WTAPLP2), WT/APP^α/α ^(= WTAPPsa), APP^α/α^/APP^-/- ^(= APPsaAPP). Click here for file

Additional file 3 **Result from the GO term and pathway analysis using DAVID for WT/APP^-/-^, WT/APP^α/α^, WT/APLP2^-/-^**. Annotation clusters are displayed with their respective enrichment score. For each annotation cluster, the gene groups (GO terms, pathways) that belong to the cluster are listed including test statistics and genes in the gene group that were significant in the respective pairwise comparison (WT/APP^-/- ^(= WTAPP), WT/APP^α/α ^(WTAPPsa), WT/APLP2^-/- ^(= WTAPLP2)). Click here for file

Additional file 4 **Lists of probe sets in the intersections of WT/APP^-/-^, WT/APLP2^-/-^, and WT/APP^α/α^**. for each significant Affymetrix probe set identifier, information about the gene (ENTREZ gene ID, gene symbol, gene name), the output from the SAM (score, numerator, denominator, fold change, q-value), and the respective pairwise comparison are displayed for the intersection of WT/APP^-/- ^and WT/APLP2^-/- ^(= WTAPP & WTAPLP2) as well as the intersection of WT/APP^-/- ^and WT/APLP2^-/- ^and WT/APP^α/α ^(= WTAPP & WTAPLP2 & WTAPPsa). Data was ranked by gene symbols. Click here for file

Additional file 5 **Lists of probe set in the intersections of WT/APP^-/-^, WT/APP^α/α^, APP^α/α^/APP^-/-^**. for each significant Affymetrix probe set identifier, information about the gene (ENTREZ gene ID, gene symbol, gene name), the output from the SAM (score, numerator, denominator, fold change, q-value), and the respective pairwise comparison are displayed for the intersection of WT/APP^-/- ^and WT/APP^α/α ^(= WTAPP & WTAPPsa) as well as WT/APP^-/- ^and APP^α/α^/APP^-/- ^(= WTAPP & APPsaAPP) and WT/APP^α/α ^and APP^α/α^/APP^-/- ^(= WTAPPsa & APPsaAPP). Data was ranked by gene symbols. Click here for file

Additional file 6 **Lists of significant probe sets of animals with mixed genetic background compared to backcrossed animals**. Results from the pairwise significance analyses: WT(R6)/APLP2(R1)^-/- ^(= WT(R6)APLP2(R1)), APLP2(R6)^-/-^/APLP2(R1)^-/- ^(= APLP2(R6)APLP2(R1)). For each significant Affymetrix probe set identifier, information about the gene (ENTREZ gene ID, gene symbol, gene name) and the output from the SAM (score, numerator, denominator, fold change, q-value) are displayed. Data was ranked by the absolute test score. Click here for file

Additional file 7 **Lists of probe set in the intersections of WT(R6)/APLP2(R6)^-/-^, WT(R6)/APLP2(R1)^-/-^, APLP2(R6)^-/-^/APLP2(R1)^-/-^**. for each significant Affymetrix probe set identifier, information about the gene (ENTREZ gene ID, gene symbol, gene name) and the output from the SAM (score, numerator, denominator, fold change, q-value), and the respective pairwise comparison are displayed for the intersection of WT(R6)/APLP2(R1)^-/- ^and APLP2(R6)^-/-^/APLP2(R1)^-/- ^(= WTAPLP2(R1) & R6R1), WT(R6)/APLP2(R6)^-/- ^and APLP2(R6)^-/-^/APLP2(R1)^-/- ^(= WTAPLP2 & R6R1), and WT/APLP2^-/- ^and WT/APLP2(R1)^-/- ^(= WTAPLP2 & WTAPLP2(R1)). Data was ranked by gene symbols. Click here for file
