# Supplementary figures and images for: Comparative transcriptome profiling of amyloid precursor protein family members in the adult cortex
Source: BMC Genomics. 2011 Mar 24;12:160. doi: 10.1186/1471-2164-12-160 (PMC3080314; doi:10.1186/1471-2164-12-160)

color key: normalized expression value

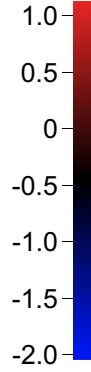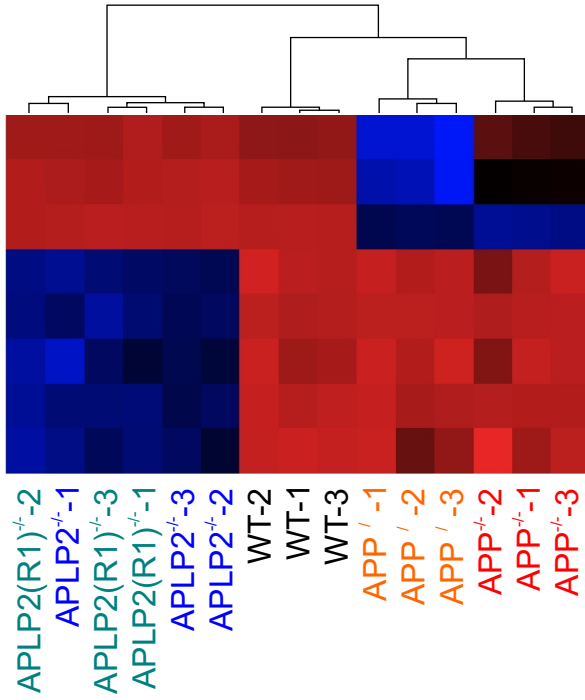

1440153\_at

1427442\_a\_at

1420621\_a\_at

1421888\_x\_at

1423739\_x\_at

1421887\_a\_at

1421889\_a\_at

1432344\_a\_at

*App*

*Ap1p2*

Supplement: Additional file 1 — Heatmap of the processed dataset. The heatmap shows the clustering of the processed data by App/Aplp2-specific probe sets. App and Aplp2 probe sets were taken from the ENSEMBL database and remapped onto the modified respective genomic loci of APP-/-, APPα/α, and APLP2-/- animals. Only probe sets that map to exonic sequences or UTRs were chosen for hierarchical cluster analysis. The first three probe sets correspond to App probe sets whereas the last five are Aplp2-specific. The values of the heatmap are normalized expression values with red and blue color representing the number of standard standard deviations above or below the mean expression for each probe set, respectively. [file 1471-2164-12-160-S1.PDF]
